# Lifespan development of attentiveness in domestic dogs: drawing parallels with humans

**DOI:** 10.3389/fpsyg.2014.00071

**Published:** 2014-02-07

**Authors:** Lisa J. Wallis, Friederike Range, Corsin A. Müller, Samuel Serisier, Ludwig Huber, Virányi Zsó

**Affiliations:** ^1^Clever Dog Lab, Messerli Research Institute, University of Veterinary Medicine Vienna, Medical University of Vienna and University of ViennaVienna, Austria; ^2^Department of Cognitive Biology, University of ViennaVienna, Austria; ^3^Royal Canin Research CenterAimargues, France

**Keywords:** attention, lifespan, development, aging, attentional control, learning, clicker training, dog

## Abstract

Attention is pivotal to consciousness, perception, cognition, and working memory in all mammals, and therefore changes in attention over the lifespan are likely to influence development and aging of all of these functions. Due to their evolutionary and developmental history, the dog is being recognized as an important species for modeling human healthspan, aging and associated diseases. In this study, we investigated the normal lifespan development of attentiveness of pet dogs in naturalistic situations, and compared the resulting cross-sectional developmental trajectories with data from previous studies in humans. We tested a sample of 145 Border collies (6 months to 14 years) with humans and objects or food as attention attractors, in order to assess their attentional capture, sustained and selective attention, and sensorimotor abilities. Our results reveal differences in task relevance in sustained attentional performance when watching a human or a moving object, which may be explained by life-long learning processes involving such stimuli. During task switching we found that dogs’ selective attention and sensorimotor abilities showed differences between age groups, with performance peaking at middle age. Dogs’ sensorimotor abilities showed a quadratic distribution with age and were correlated with selective attention performance. Our results support the hypothesis that the development and senescence of sensorimotor and attentional control may be fundamentally interrelated. Additionally, attentional capture, sustained attention, and sensorimotor control developmental trajectories paralleled those found in humans. Given that the development of attention is similar across humans and dogs, we propose that the same regulatory mechanisms are likely to be present in both species. Finally, this cross-sectional study provides the first description of age group changes in attention over the lifespan of pet dogs.

## INTRODUCTION

One of the most intensely studied cognitive processes in humans and animals is attention: the ability to selectively process one aspect of the environment over others. Attention is pivotal to perception, consciousness and will (Washburn and Taglialatela, 2006). In humans the different components of executive control (including attentional control) develop at different ages, and follow a quadratic relationship with age over the lifespan; increasing in power, speed and complexity from infancy to young adults, and declining differentially in old age depending in part on the brain areas involved (Craik and Bialystok, 2006).

Attention has been proposed to consist of multiple components that interact during cognitive functioning (Cornish et al., 2006). One model, which clearly delineates the separate components of attention, is Sohlberg and Mateer’s (2001) hierarchical clinical model of attention. The model was originally based on the recovery of attentional processes of brain damaged patients after coma, and details five components of attention recruited in tasks of increasing difficulty: focused, sustained, selective, alternating, and divided attention. Each separate component of attention has been extensively studied in humans, which has led to the discovery of different effects of age on the development of attention. For example, age has little influence on orienting to a single location (Enns and Cameron, 1987), and adult efficiency is already reached at 5–7 years of age (Michael et al., 2013). There was also little influence of age on simple sustained attention measures over short periods (Giambra and Quilter, 1988; Berardi et al., 2001). Performance in alternating attention (task switching) and selective attention tests depends on an individual’s level of executive attentional control, and crucially involves active inhibition (Cepeda et al., 2001). Both have been found to follow a U shaped developmental trajectory in humans, with abilities peaking in the 20- to 30-year-old age groups (Cepeda et al., 2001; Clark et al., 2006). One other important additional component of cognitive development and decline which could affect attentional abilities is age-related changes in sensory and motor processes. In a cross-sectional lifespan study, Clark et al. (2006) found that two measures of sensorimotor abilities of humans followed quadratic age trends, with performance peaking at the 20–39 years middle age range.

Except in humans, rats and some non-human primates, studies that incorporate measurements of the separate components of attention and sensorimotor control over the lifespan are lacking in mammals and birds. Since attention is a complex cognitive process, and the effect of aging varies with the different aspects of attention investigated, comparative lifespan studies can help to clarify and confirm the main findings in the human literature (Macphail, 1987). Non-human mammals have the same general patterns of development and decline of cognitive functions as humans (Pearce, 2008) and can provide good models for the development and aging of specific cognitive domains. From previous studies we know that attention operates in non-human mammals in much the same way as it does in humans (Blough, 2006). However, the few studies on the development of attention in non-human mammals provide limited knowledge for four reasons: (1) they have focused solely on tests that require extensive training amounting to weeks, months, or even years of testing: such as selective attention performance and response latencies in discrimination learning or matching tests, and thus did not attempt to measure the array of components which constitute attention. (2) Many have failed to provide an adequate sensorimotor control. (3) They tested only lab animals, and of those, (4) small sample sizes with only few age groups were used (Bartus et al., 1979; Presty et al., 1987; Rapp, 1990; Adams et al., 2000; Schoenbaum et al., 2002). Despite of these limitations, the laboratory beagle, on which the majority of studies examining age differences in dogs have focused, has been recognized as a useful animal model, since their measures of learning, memory, and executive function decline with age, similarly to humans (Tapp et al., 2003).

On the other hand, the classic paradigms originally developed for examining attention in humans have so far rarely been used on pet dogs (selective attention: Mongillo et al., 2010; sustained attention: Range et al., 2009b), though this would allow for better comparisons with humans over the lifespan. Even fewer studies have carried out direct comparisons between laboratory dogs and humans in tasks involving attention, and their results are not conclusive. For instance, in the study of Boutet et al. (2005), dogs showed significant age-dependent deficits, but results from the human sample revealed no age effects.

In contrast to dogs, rodents and primates kept in laboratories, pet dogs present useful subjects for several reasons. Pet dogs are not only available in a great numbers, but they also share an evolutionary and developmental history with humans due to domestication. Dogs can be tested in their natural environment that they share with humans, often using the same observations and experimental protocols (Miklósi et al., 2004). Increasingly the dog is being recognized as an important species for modeling healthspan and longevity, aging and associated diseases such as Alzheimer’s disease (Opii et al., 2008), and psychiatric disorders, such as human obsessive–compulsive disorder (Rapoport et al., 1992) and attention deficit hyperactive disorder (Lit et al., 2010) due to the fact that dogs share the same challenges in their daily lives as humans. Despite being distantly related genetically, the fact that pet dogs have evolved in a human-dominated environment may have led to the development of similar social behavior to humans (Hare and Tomasello, 2005), which increases the probability that dogs and humans may share some of the same brain mechanisms (Miklósi et al., 2007). The high genetic variability and differing environmental experiences found in pet dogs provides the foundation for individual differences and personality (Jones and Gosling, 2005), and can contribute to a more realistic picture of development and aging of cognition. In contrast, animals kept in standardized laboratory conditions are often from highly inbred lines, with limited social and environmental experience.

Finally, from an applied perspective, studying lifespan development of attentiveness is particularly relevant for dogs, since a large proportion of the general public lives and interacts with dogs on a daily basis (Coren, 2012). The extent to which a dog can concentrate selectively on specific aspects of the environment and to exclude others is of utmost importance for effective training, social learning, and communication; all of which rely crucially on a dogs’ ability to maintain attention toward humans (Lindsay, 2001; Range et al., 2009a).

The majority of studies examining cognitive abilities in pet dogs have used cross-sectional designs, by examining just a few age groups. Such studies give little information on how task performance develops with age. Cross-sectional studies can be used to indicate developmental change by allowing trajectories to be mapped from individuals at different developmental stages (Thomas et al., 2009). They cannot replace longitudinal studies however; one major concern is that there is no guarantee that behavior on the same test is being driven by the same processes at different ages. Nevertheless, cross-sectional studies provide valuable information as they can form the basis to design subsequent efficient longitudinal studies (Kraemer et al., 2000). The importance of robust methods when using developmental trajectories in cross-sectional studies has been recently emphasized (Thomas et al., 2009). The use of the trajectory method to study developmental relations is possible wherever there is a wide age range in the sample, and as long as the influence of outliers, or the presence or absence of ceiling and floor effects are checked. The cross-sectional method commonly used begins by constructing a trajectory for each attentional measure across normally aging individuals at different ages. In subsequent studies, the trajectories of groups suffering from canine cognitive dysfunction (CCD) or attention deficits can be compared to this reference by linking changes in performance to chronological age, and establishing whether impairments exist (Annaz et al., 2010), and the cross-sectional studies can be followed up by longitudinal studies to corroborate the data.

The goals of the present study were to (1) develop attention tests, which can be used to examine the effects of development and aging (but do not require extensive training), by adapting simplified versions of tests from the human literature, (2) investigate the normal rate of attention development and decline in a cross-sectional sample of pet dogs ranging in age from 6 months to old age, (3) compare the basic developmental trajectories of the different sub-processes of attention and sensorimotor control in humans using results from previous studies, with the present results found in pet dogs. Compiling cross-sectional data from the majority of the dogs’ life course will allow us to examine normative change, which occurs when individuals change in a similar way during a specific period within the life course (McCrae et al., 2000). For these purposes we tested dogs with humans and with objects or food as attention attractors in two separate experiments in order to assess their attentional capture, sustained attention, selective attention, and their sensorimotor abilities.

## GENERAL METHODS

### SUBJECTS

One hundred and forty five dog-owner dyads participated in this study. Dog ages ranged from 6 months to 13 years and 10 months (**Table 1**). All recruited dogs were Border collies to exclude effects of breed differences. Owners could participate with more than one dog, therefore there were more dogs than owners (*N* = 122). There were more female than male owners, (*F* = 108, *M* = 14) and owners were aged between 12 and 72 years. Recruitment was concluded on the completion of seven age groups (**Table 1**). The choice of the age groups aimed to reflect the developmental periods in the Border collie [late puppyhood, adolescence, early adulthood, middle age, late adulthood, senior, and geriatric (Siegal and Barlough, 1995)].

**Table 1:** Age, sex, and reproductive status of subjects.

Age group	Life stage	Age in years	Mean + SD age in years	Male (neutered)	Female (neutered)	Total
Group 1	Late puppyhood	0.5–1	0.83 + 0.11	10 (0)	13 (1)	23
Group 2	Adolescence	>1–2	1.51 + 0.32	10 (2)	13 (2)	23
Group 3	Early adulthood	>2–3	2.54 + 0.32	9 (4)	10 (3)	19
Group 4	Middle age	>3–6	4.62 + 0.89	9 (4)	12 (5)	21
Group 5	Late adulthood	>6–8	7.13 + 0.63	13 (7)	8 (8)	21
Group 6	Senior	>8–10	8.88 + 0.57	10 (5)	9 (9)	19
Group 7	Geriatric	>10	11.61 + 1.03	8 (6)	11 (11)	19
Total				69 (28)	76 (39)	145

All dogs were tested in the “Vienna Canine Cognitive Battery” (Wallis et al., in preparation), of which the attention tests used for this study were a part. The dogs had visited the lab on a minimum of three occasions before the attention testing, and all had prior experience of working with the experimenter.

Owners filled in an extensive demographic questionnaire to obtain details on their dog’s training experience including 13 different training types. Puppy school (83% participated), basic obedience (68%), high level obedience (49%), Protection training (3%), agility (70%), search and rescue training (6%), companion dog training (31%), dog dancing/trick training (54%), dummy training (11%), nose work (27%), sheep dog training (52%), therapy dog (13%) and other (22%). On average, dogs participated in five different training types. Dogs scored according to attendance: no experience = 0, sporadic training = 1, once or twice a month = 2, once or twice a week = 3, and completed training (with or without an exam) = 4. Individual scores in each type of training were added up to a maximum of 52 points. Training score was correlated with age in months (Spearman’s *rho* = 0.458, *p* = < 0.001), therefore in all models, training score and age were analyzed separately. To take into account the dogs’ current training participation, the average number of training hours per week was calculated for each dog. This calculation was made based on its current training schedule when the cognitive battery was performed. Mean training hours per week was 5.6 ± 4.49, (range from 0 to 25 h) and was negatively correlated with age in months (Spearman’s *rho* = -0.272, *p* = 0.001). However, training score and current training hours were not correlated (Spearman’s *rho* = 0.016, *p* = 0.394).

### CRITERIA FOR EXCLUSION OF SUBJECTS

To be included in the study, dogs were required to meet specific criteria. Owners filled in information about their dogs’ recent medical care, disease history, and whether their dogs were currently on any medication. Dogs which were not medically fit [including dogs which suffered from eye abnormalities or second stage (visible) cataracts] were excluded, or testing was postponed until they were in normal health (testing of one dog was postponed due to false pregnancy, another due to actual pregnancy). Owners of dogs older than 6 years also filled in a CCD questionnaire [translated into German, based on Salvin et al. (2011a)]. None of the dogs showed significant behavioral signs of CCD (according to the CCD rating scale; all scored under 50 points). Only three dogs had to be excluded: one because of video recording malfunction, and two because of medical problems.

### TEST SETTING

All tests were conducted in an experimental room (5 m × 6 m) by the same experimenter who was blind to the age of the subjects. In the testing room two doors were located approximately 2 m apart on one wall. The only furniture present was a small table standing next to the side wall and a chair for the owner.

### DATA COLLECTION AND STATISTICAL ANALYSIS

Tests were videotaped using a set-up of four digital video cameras, which were connected to a video station outside of the testing room. Videos were analyzed with Solomon Coder beta 12.09.04 (Copyright © 2013 by András Péter) using a continuous sampling technique. Statistical analyses were performed in R 3.0.1 (R Core Team, 2013). Separate statistical models were calculated first with age as a continuous variable (we tested for linear and/or quadratic relationships), and then with age as a categorical variable to look for specific differences between age groups. Separate models were also calculated to assess the effects of training score and current training hours. Normality and homoscedasticity were assessed via residuals’ distribution charts and plots of residuals against fitted values. Non-significant predictors (*p* > 0.05) were removed from the model, and are not reported in the results. Results are presented as mean ± standard deviation unless otherwise indicated. To analyze the effect of outliers, variables were converted to standard *z* scores, any outliers of *z* scores of greater than ±3 were removed from the analysis, and the models re-run.

## EXPERIMENT 1: ATTENTIONAL CAPTURE AND SUSTAINED ATTENTION

In experiment 1 we tested whether dogs’ attentional capture and their sustained attention differed by age in two different contexts, Event 1 comprised of a moving object, and Event 2 a moving human and object. Previous research on attention in monkeys using a touch screen by Baxter and Voytko (1996) determined that attentional capture was preserved in aged rhesus monkeys. Zeamer et al. (2011) compared sustained attention in healthy young and aged rhesus monkeys, using a continuous performance task (individuals were trained to respond to one of three stimuli by touching a screen). Results showed that aged animals made significantly more errors than young animals. This task took many trials to learn before testing could take place. Therefore, for this experiment we simplified the sustained attention test by removing the need for a trained behavioral response to indicate attention. Instead we measured dogs’ attention to two stimuli, as indicated by time spent with the head (used as a proxy for gaze direction) directed toward the stimuli.

Previous studies focusing on measures of attention to novelty in dogs and rats found that exploratory behavior varied significantly with age; with older subjects showing the lowest levels of sustained attention (Soffié et al., 1992; Handa et al., 1996; Siwak et al., 2001; Rosado et al., 2012). Therefore, based on the previous research cited above, we predicted that dogs would show no age differences in attentional capture, and sustained attention to the two stimuli was expected to decline with age.

### METHODS

#### Test setting and procedure

At the beginning of the experiment, the owners entered the experimental room with their dog on a leash. A hook on the wall next to a window allowed dogs to be tethered in one location. The owners attached their dogs to the 1.5 m leash on the hook, and sat down on a chair facing away from the dog toward the window. They started to fill in a questionnaire on an iPad. Owners were instructed to ignore their dog and the actions of the experimenter, and to be quiet and still. All owners followed guidelines, and did not attempt to interact with their dogs. Two conditions were presented in a counterbalanced order to each dog, Events 1 and 2.

**FIGURE 1:**
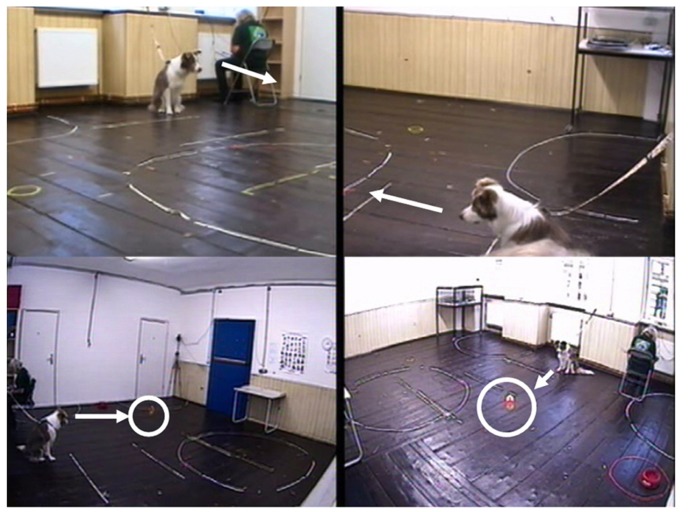
**Still video frame from set-up of experiment 1 – Event 1 condition**.

***Event 1:*** After the dog and owner were in position, the experimenter pulled a fishing line, which was attached to a small orange plastic watering can (child’s toy) placed in the center of the experimental room. The line ran through a metal hoop in the ceiling in the testing room, allowing the object to be manipulated by the experimenter from outside the room. The object was moved up and down in front of the dog (but the dog was prevented from approaching it by the leash) for approximately 1 min (**Figure 1**). After this time the experimenter fixed the toy to the ceiling and a tone indicated that the owner and dog should leave the room.

***Event 2:*** After the dog and owner were in position, the experimenter entered the testing room, closed the door, walked to the wall opposite the dog, and proceeded to walk up and down the length of the wall (6 m) pretending to paint the wall with a roller with her back to the dog. The experimenter removed her shoes before the test, and walked as quietly as possible. At no point did the experimenter gain eye contact with the dog. After 1 min the experimenter left the room, and a tone indicated that the owner and dog should leave the room.

#### Data collection and statistical analysis

We used the latency to orientation [LO; measured from the first detectable movement of the toy/door handle up to the point where the dogs gaze (head and nose) was centered upon the stimulus (toy/door opening/human entering)] as a measure of attentional capture, and the average gaze (AG)-bout duration (total duration looking time divided by frequency of looks), and the percentage of total looking time (PTLT) as measures of sustained attention. Dogs that were already orientated to the stimuli when the stimuli were first presented, were excluded from the LO analysis (Event 1: *N* = 24, Event 2: *N* = 13). A randomly chosen set of 20 dogs were double coded independently by two coders, and inter-observer reliability for LO, AG, and PTLT was excellent (*r* > 0.89, *p* <0.001 for each variable).

Latency to orientation was inverse-transformed, AG was log-transformed, and PTLT was square-transformed to attain homogeneity of variances, and additionally we fitted a variance structure which allowed for variance to differ between the two conditions (constant variance). Data was analyzed using linear mixed effects models (LMMs; Pinheiro and Bates, 2000) with condition (Event 1 vs. Event 2), age and experiment order (Event 1 first vs. Event 2 first) as fixed effects and dog identity as a random factor. Additionally, the potentially confounding variables sex and neuter status were included as fixed effects. After testing for age effects we then re-ran the model with training score and current training hours as fixed effects and dog identity as a random factor. We included the two-way interaction between condition and age, training score or current training hours respectively to test whether any effects may be restricted to one condition.

To examine whether dogs attentional performance was consistent across different contexts the relationship between PTLT at Event 1 stimulus and PTLT at Event 2 stimulus was calculated, using a Spearman’s rank correlation test.

### RESULTS

**FIGURE 2:**
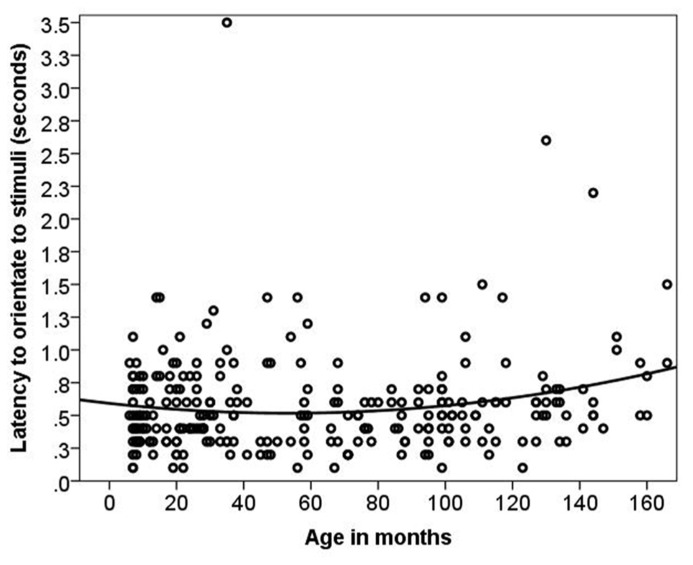
**The quadratic relationship between latency to orient and age in months**.

Dogs’ LO to the stimulus was on average 0.57 s (range = 0.1–3.5 s, SD = 0.38 s). The relationship between age and LO was best described by a quadratic function [LMM, *F*(1,141) = 4.97, *p *= 0.01, **Figure 2**]. When using age group as a predictor no significant age differences or interactions were found (*p* = 0.28). There was no significant difference in LO to Event 1 vs. Event 2 stimuli. The removal of two outliers did not change the results.

**FIGURE 3:**
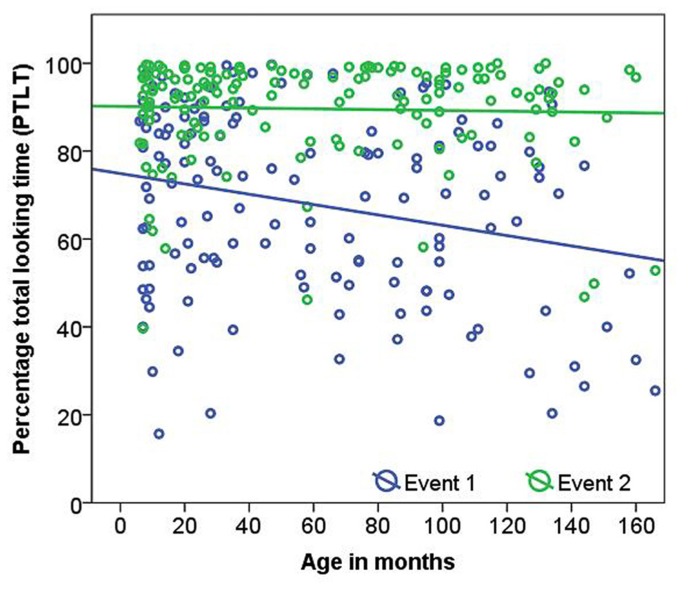
**Age related changes in percentage total looking time (PTLT) to Event 1 and Event 2 stimuli**.

Percentage total looking time was significantly higher for Event 2 than for Event 1 (Event 1 = 66.17 ± 22.13; Event 2 = 90.43 ± 10.86; LMM, *F*(1,140) = 221.01, *p* <0.001). There was a significant interaction between condition and age in months [LMM, *F*(1,140) = 5.35, *p* = 0.02, **Figure 3**]. PTLT decreased with age in Event 1 (Spearman’s rho = -1.98, *p* = 0.02) but not in Event 2 (Spearman’s rho = 0.042, *p* = 0.62). When comparing age groups no significant age differences or interactions were found. When three outliers were removed all reported results remained significant.

Average gaze-bout length was longer in Event 2 than in Event 1 [Event 1 = 12.51 ± 11.70; Event 2 = 42.82 ± 30.89; LMM, *F*(1,141) = 289.03, *p* < 0.001]. There were no significant effects of age on AG.

With both variables (PTLT and AG), attention paid to Event 1 was significantly positively correlated with attention paid to Event 2 (PTLT: Spearman’s *rho* = 0.224, *p* = 0.010; AG: Spearman’s *rho* = 0.270, *p* = 0.001). These results remained significant after removing outliers.

Training score and current training hours had no significant effects on any of the variables measured.

### DISCUSSION

When examining dogs’ attentional capture abilities across age a significant quadratic relationship was found. Age differences can possibly be explained by a slight sensory motor decline in the aged dogs, and a heightened sensitivity to sound/movement in the middle aged dogs. However since latencies to orientation did not differ in Event 1 and Event 2 conditions, and response latencies in the senior age group were not significantly different from the other age groups, the observed relationship was minimally effected by age. Regardless of the original orientation of the dog, all dogs very quickly orientated to the stimuli in Event 1 and 2, and we conclude that the physiological condition of the dog minimally affected its ability to orientate its gaze to the stimuli.

Measures of sustained attention were expected to decline with age in both conditions. However, only attention to the Event 1 stimulus showed a significant reduction with age in accordance with our predictions. The novel stimulus and strange movement of the inanimate object generally caused a startle response in the dogs, and an increase in frequency of looks to the stimulus compared to Event 2. The older dogs showed a decrease in overall looking time compared to young dogs, which could be explained by a life-long learning process to reduce reaction to novel external stimuli, such as moving objects (children, cars, bicycles, etc.). Dogs learn to attend selectively, which helps them to focus their attention on relevant stimuli (for example the owner), whilst ignoring irrelevant occurrences (Lindsay, 2001). We found no age effect on attention paid to Event 2, which may be due a ceiling effect (almost half of the dogs paid attention to the stimuli for over 95% of the time). Therefore, the interaction found between age and stimulus type may be an artifact of the ceiling effect. Future studies will need to determine whether sustained attention toward a social type stimulus might also decrease with age, for example by increasing the duration of presentation of the stimulus in Event 2. Here we can conclude that even senior dogs are capable of high levels of sustained attention over 1 min if the stimulus is of high relevance to them.

Percentage total looking time and AG-bout duration was found to be higher in Event 2 (experimenter painting the wall) than in Event 1 (moving plastic watering can). One possible explanation for this difference is that the size of the stimuli caused a bias in attentional allocation. The type of movement (vertical vs. lateral), the distance of the stimuli from the dog, and the novelty of the stimulus could also have influenced the dogs’ attention. Previous studies have indicated that dogs prefer to attend to novel objects over familiar ones (Kaulfuss and Mills, 2008) and also to novel human faces when compared to familiar faces (Racca et al., 2010), therefore we might have expected dogs to attend to Event 1 and 2 similarly. A main difference between the two event situations was that Event 1 contained a non-social stimulus and Event 2 a social stimulus. It seems likely that positive experiences with the experimenter gained in the previous tests of the test battery could have motivated the dogs to attend to her, over the novel non-social object. Horn et al. (2013) found that the nature of past interactions with a human specifies the dogs’ relationship with them, and increases attention to that person. Positive reinforcement during previous training experiences has been found to be highly correlated with levels of attention (Lindsay, 2001). Therefore reinforcement of attention in one situation should improve attending to the same stimulus in different contexts.

In sum, by the age of 6 months, Border collie attentional capture and sustained attentional abilities were already at adult levels, which is comparable to the finding of similar tests in human subjects (Berardi et al., 2001; Michael et al., 2013). Nevertheless, individual differences occurred consistently across the different contexts (i.e., dogs which looked longer at the Event 1 stimulus also looked longer at the Event 2 stimulus) which could be a consequence of an underlying personality trait.

## EXPERIMENT 2: SELECTIVE ATTENTION

In experiment 1 we found minimal age effects on attentional capture and sustained attention in pet dogs. Previous studies have established that increasing task difficulty enhances the likelihood of finding age related differences in humans (McDowd and Craik, 1988), therefore we performed a second experiment, where we measured whether dogs selective attention and sensorimotor abilities differed by age during task switching.

One common method widely used to assess selective attention is the visual search task, which requires participants to attend to a target stimulus while disregarding irrelevant “distracter” information. Previous studies have shown that senior dogs are significantly impaired in accuracy and reaction time compared to younger animals in a visual search task with distracters (Snigdha et al., 2012). In a social version of this task, Mongillo et al. (2010) simultaneously presented the owner and a stranger to the dog, forcing it to be selective as to whom it observed. Older dogs discriminated between the owner and the stranger to a lesser extent, because they oriented longer to the stranger compared to adult dogs. Similarly, age and stimulus relevance have a strong influence on selective attention also in humans (Hommel et al., 2004) and non-human primates (Zeamer et al., 2011).

Previous studies examining sensorimotor control in non-human animals, have found a significant decline with age, as in human studies. For example, Wallace et al. (1980) discovered that tasks requiring coordinated control of motor and reflexive responses in rats (such as descent of a wire mesh pole) showed significant declines with age in four age groups (6, 12, 18, and 24 months). In their study of normative behavioral changes associated with “successful aging” in dogs, Salvin et al. (2011b) found that difficulty in finding food increased significantly across three age groups (<10, 10–12, >12 years). This could reflect alterations in the cognitive processing of sensory information, or could be a result of physical deterioration of the visual, audio, or olfactory organs. Therefore it is necessary to exclude physical degeneration as the cause of apparent changes in cognition.

In Experiment 2 we investigated whether dogs differ by age in their selective attention when switching between two tasks: finding food on the floor, and gaining eye contact with the experimenter. Additionally we examined whether dogs differed by age in their ability to find dropped food (sensorimotor performance). Based on human and animal studies, we predicted that younger and older dogs would show an impaired performance in selective attention and sensorimotor control, producing a quadratic effect with age.

### METHODS

#### Test setting and procedure

For this experiment, the owner sat positioned at the back wall of the experimental room and filled in a questionnaire. The experimenter stood in the center of the room facing the owner, holding a clicker in her right hand, and the other hand was free. Both hands were positioned in a relaxed posture by her sides. The experimenter had a food pouch on her belt, positioned at her back. Sausage, which had been cut into <1 cm^3^, was used as a food reward. For the first trial, the experimenter called the dog to her, and threw a piece of sausage on the floor in front of her for the dog to find. She then remained motionless until the dog established eye contact with her, whereupon she immediately clicked the clicker, took a piece of food from a pouch on her belt, tossed the food on the floor to the left or the right of the dog, and then waited for the dog to establish eye contact again after it found and ate the food. The sausage was always thrown so that the dog had to move out of its current position to obtain the food. If the dog wandered further than 2 m from the experimenter, and no longer showed interest, the experimenter rustled the plastic bag containing the sausage, and then returned to her position, with arms and hands at her sides. The experimenter continued this task for a total of 5 min.

We considered this experiment to be demonstrative of dogs’ selective attention abilities, as the dogs had to change their focus of attention in the presence of competing stimuli: the experimenter’s hand which moments ago threw a piece of sausage, the floor where food could be found, and the face of the experimenter (for which the dog was rewarded when looking at). Thus in this task the dog had to disregard (inhibit) irrelevant “distracter” information in order to receive the food reward.

#### Data collection and statistical analysis

We used two parameters as measures of attention in this task: the latency to eye contact (LEC) with the experimenter (measured from the moment the dog had taken the food into its mouth until the dog looked up into the face of the experimenter, which was marked by a click from the clicker), and the latency to find food (LFF; measured from the moment the piece of sausage left the experimenters hand, until the dog found the food, and took it into the mouth). The dogs’ initial performance in the task was measured by taking the average of the first three trials in both LEC and LFF. A randomly chosen set of 20 dogs was double coded independently by two coders and inter-observer reliability for LEC and LFF was excellent (*r* > 0.87, *p* < 0.001 for each variable). LEC was log-transformed, and LFF was inverse-cube transformed to attain homogeneity of variances. The data was analyzed using linear models (LMs; Chambers, 1991), with age and previous clicker experience (yes/no) as fixed effects. Forty three percent of the subjects were clicker trained, the proportion of clicker trained dogs was highest in age group 1, lowest in age group 7, and clicker training was weakly correlated with age in months (Spearman’s *rho* = -0.191, *p* = 0.021). Additionally, the potentially confounding variables sex and neuter status were included as fixed effects. Age group comparisons were analyzed using LMs with generalized least squares (GLS; Davidian and Giltinan, 1995) and a variance structure which allowed for variance to differ between age groups was fitted. After testing for age effects we then re-ran the models with training score and current training hours as fixed effects.

Learning across trials was examined by taking the first 20 trials of LEC and LFF for all dogs (seven dogs were removed from the analysis as they did not complete 20 trials within the 5-min period). LEC learning data was inverse square-root transformed, and LFF data inverse log transformed. To obtain homoscedasticity of data, we also fitted a variance structure which allowed for variance to differ with trial number (exponential variance), and between age groups (constant variance). Data was analyzed using LMM (Pinheiro and Bates, 2000), with age as categorical variable (seven age groups), trial number, previous clicker experience (yes/no), sex, and neuter status, included as fixed effects. We then re-ran the models with training score and current training hours instead of age as fixed effects. We included the two-way interaction between trial number and age, clicker experience, training score or current training hours respectively to test whether learning differed between age groups, or with clicker experience, training score or current training hours.

Finally, to examine whether dogs attentional performance was consistent across different contexts, the relationship between LEC and LFF was analyzed using a Spearman’s rank correlation test.

### RESULTS

#### Initial latencies

**FIGURE 4:**
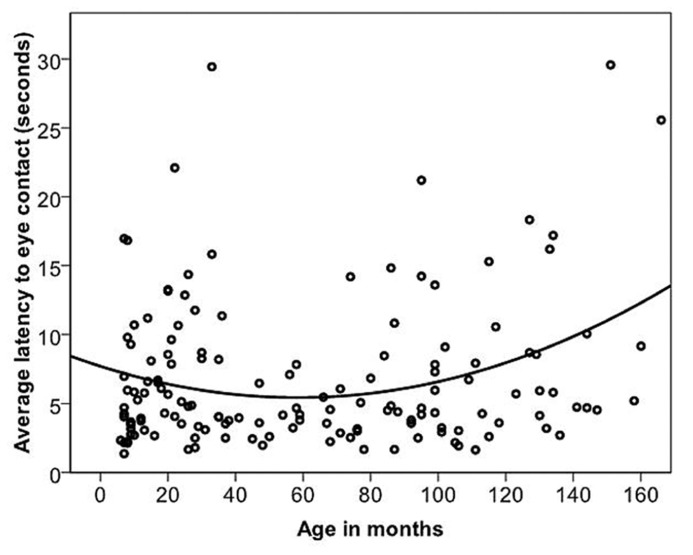
**The quadratic relationship between the average latency of the first three trials of individual dogs to gain eye contact with the experimenter and age in months**.

Dogs’ LEC with the experimenter was on average 6.82 s

(range = 1.37–29.57 s, SD = 5.34 s). The relationship between age in months and LEC was best described by a quadratic function (**Figure 4**; **Table 2**). Previously clicker trained dogs were faster to gain eye contact than non-clicker trained dogs (**Table 2**). When comparing the latencies in the age groups, performance peaked in group four (middle aged: 3- to 6-year-olds). Therefore, we compared all other age groups to group four to look for differences in performance. There was a significant difference found between the age groups [GLS, *F*(6,145) = 3.99, *p* = 0.001]. LEC was significantly higher in age groups two, three and seven compared to age group four (*t *> 2.04, *p* = <0.05). However, when three outliers were removed, the quadratic relationship between age in months and LEC was no longer significant, and LEC was significantly higher only in age groups two and seven, when compared to age group four. 

**Table 2:** Factors affecting initial latency to eye contact (LEC) and latency to find food (LFF).

Dependent variable	Model term	DF	Sample size	*F*-value	*p*-Value
LEC	Age (linear)	1	142	2.353	0.127
	Age (quadratic)	1	142	4.395	0.038*
	Clicker experience	1	142	4.604	0.033*
LFF	Age (linear)	1	143	0.943	0.333
	Age (quadratic)	1	143	5.723	0.018*
	Clicker experience	1	143	1.085	0.299

^*^p < 0.05.

**FIGURE 5:**
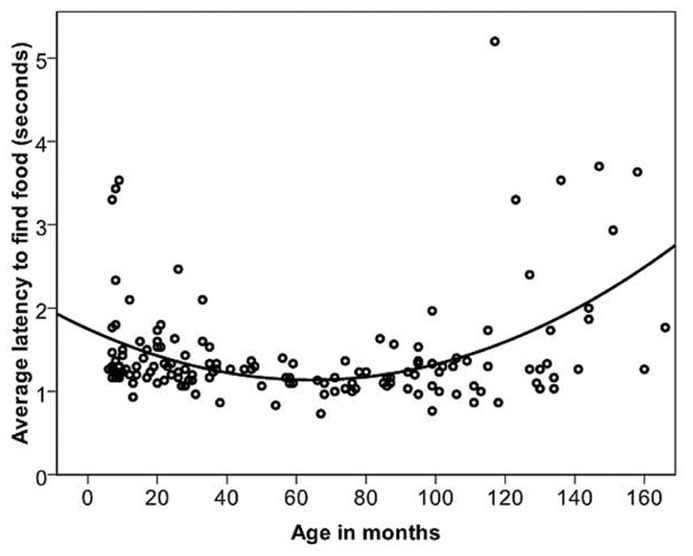
**The quadratic relationship between the average latency of the first three trials of individual dogs to find food and age in months**.

Dogs’ LFF was on average 1.45 s (range = 0.73–5.2 s, and SD = 0.65 s). The relationship between age in months and LFF was best described by a quadratic function (**Table 2**; **Figure 5**). When comparing the latencies in the age groups, performance again peaked in group four (middle aged). We compared all other age groups to age group four and found that there was a significant difference between age groups [GLS, *F*(6,145) = 5.53, *p* = <0.001]. LFF in age groups one, two, three and seven was significantly higher than LFF in age group four (*t* <-2.63, *p* = <0.01). After removing five outliers, all effects found remained significant.

Latency to eye contact was significantly positively correlated with LFF (Spearman’s *rho *= 0.232, *p* = 0.005). Training score and current training hours had no significant effects on any of the variables measured.

#### Learning across trials

Results from the learning across trials analysis produced a significant effect of trial number on LEC, indicating that individuals improved in gaining eye contact over trials (**Table 3**). There was also a significant trial number by age group interaction (**Table 3**). When compared with the top performing age group in the initial trials (group four), group two (1- to 2-year-olds) showed a significantly steeper learning curve. Clicker experienced dogs showed a tendency toward shorter latencies to eye contact than dogs with no clicker experience.

**Table 3:** Linear mixed effects models factors affecting learning across trials in latency to eye contact (LEC) and latency to find food (LFF).

Dependent variable	Model term	DF	Sample size	*F*-value	*p*-Value
LEC	Trial number	1	2614	221.120	<0.001*
	Clicker experience	1	130	3.475	0.065
	Age group	6	130	1.582	0.142
	Trial number: age group	6	2614	4.298	<0.001*
LFF	Trial number	1	2620	0.540	0.464
	Clicker experience	1	129	7.530	0.007*
	Neuter status	1	129	7.070	0.009*
	Age group	6	129	5.570	<0.001*

^*^p < 0.05.

As we found a significant interaction between age and trial number, we then controlled for dogs initial performance in this task, which could influence the rate of learning, by running LM using LEC as the response variable and trial number as a fixed effect, to obtain regression slopes for each individual. We then ran LMs with regression slope as the response variable, initial performance (intercept of the regression) as a predictor in addition to age (seven age groups), clicker (previous experience: yes/no), sex, neuter status, training score and current training hours. Results from the model showed a highly significant effect of intercept [LM, *F*(1,128) = 67.59, *p* = < 0.001] and a tendency toward a significant difference between the age groups [LM, *F*(6,128) = 2.13, *p* = 0.054]. When comparing the age groups, the only significant result was that group two showed significantly steeper learning curves compared to group one (*t* = -2.71, *p* = 0.007).

Results from the LFF model revealed that dogs’ performance differed significantly among the age groups (**Table 3**). Age groups one, two, three, five, six, and seven had significantly higher LFF than group four (*t* > 2.00, *p* = <0.05). There was no significant effect of trial number; therefore the dogs did not significantly improve in their ability to find food over the 20 trials. However, clicker experienced dogs showed a shorter LFF than dogs with no clicker experience, and additionally, neutered dogs were quicker to find the food than intact dogs (**Table 3**).

Training score and mean number of hours spent in training per week had no significant effects on any of the variables measured.

### DISCUSSION

The dogs’ selective attention and sensorimotor abilities showed differences between cross-sectional age group means which peaked at middle age (3–6 years), when their LEC and to find food was the lowest. LFF showed a quadratic distribution with age in months, and was highly correlated with LEC. Deficiencies in LEC present in the younger (adolescent) and oldest age groups could be due to: (1) lower motivation, (2) reduced sensorimotor capability, or (3) deficiencies in attentional control. Motivational differences to attend to the experimenter in the age groups are unlikely, since in experiment 1 we found no age differences in sustained attention duration in Event 2 which included a social component, indicating that all of the age groups were equally motivated to attend to the experimenter. Also food motivation did not vary with age, as dogs’ performance in LFF over the 20 trials remained stable over time. Therefore we suggest that, due to the low range of LFF values, dogs were equally motivated to find the food and to participate in the trials.

However, there was evidence that the LFF was affected by the dogs’ sensorimotor capability. Age differences in the dogs’ initial performance were found, and remained consistent over the 20 trials. LFF provided an effective sensory and motor control, which is comparable to similar tests in humans and rats (Wallace et al., 1980; Clark et al., 2006). Given the very short latencies for finding food (mean 1.45 s), and also the LO in experiment 1 (mean 0.57 s), it is unlikely that sensorimotor deficiency explain all of the differences we found in the age groups concerning the LEC. The differences found in the adolescent and oldest age groups were most likely due to deficiencies in attentional control abilities, or increased distractibility. The results from this study complement previous research on selective attention in dogs, which point to a reduced capacity of older dogs to inhibit distracting stimuli (Tapp et al., 2003; Mongillo et al., 2010; Snigdha et al., 2012). With our experimental design we were not able to determine whether reduced visual processing speed, reduced cognitive resources (impairments in other cognitive and learning abilities), and/or an inability to ignore distracting information (decrease in performance accuracy) or a combination of these factors was responsible for the observed results. Future studies should try to separate these three functions to determine to what extent they effect development and aging of attentional processes in the dog. It is also important to note that, despite of its practical importance and relevance for social behavior, measuring social attentiveness through eye contact has an inherent constraint. Since the experimenter with whom the dogs are required to establish eye contact cannot be prevented from seeing the dogs and some of their characteristics, such as age, it is impossible to make sure that she/he treats all subjects in the same way. In our experiment, the experimenter could easily discriminate the 6 month from the 12-year-old dogs. During the clicker training for eye contact, although the task required that the experimenter remain motionless whilst waiting for the dog to take up eye contact, unconscious subtle movements by the experimenter may have inadvertently captured the dogs attention. Potentially, this effect might have contributed to some of the differences we found. Future studies should attempt to find other measurements of social attention that can control for such effects but at the same time can be as informative as mutual gaze.

In order to fully examine the lifespan development of attention, puppies as young as 2 months of age would need to be tested. Using a similar method, in a study carried out by Passalacqua et al. (2011) over 50% of puppies at 2 months of age looked at the experimenter within 1 min in an “unsolvable task” paradigm. Adult dogs (average age 4.4 years) were significantly faster to look at the experimenter when compared to 4.5 and 2 months old puppies, which suggests that human directed gazing behavior improves with age, possibly through a history of positively rewarded human interactions. In the present study only adolescent and geriatric dogs showed slower latencies. The onset of sexual maturity varies according to the speed of development of the animal, and is reached between 6 and 18 months of age depending on the breed (Miklósi, 2008). Behavioral maturation in the dog does not occur at this time: although capable of mating, dogs do not display fully adult behavior until around 2–3 years of age. Our results suggest that the maturation of selective attention may coincide with behavioral maturation in the dog. Adolescent dogs go through a hormonal surge which often affects their behavior, including their ability to pay attention and respond to previously learned cues (Lindsay, 2001). During this period an imbalance between attention and affective and motivational networks cause emotional and motivational distractors to have a detrimental effect on attentional control, which explains why adolescent behavior is often erratic (Crone, 2009).

Dogs’ selective attentional performance improved across the 20 training trials in all age groups; therefore dogs from 6 months to 14 years all showed the ability to learn, consistent with previous studies in dogs (Lillard and Erisir, 2011). In this task, even though older and adolescent dogs showed deficiencies initially, they were able to significantly reduce their latencies with training. Dogs aged from 1 to 2 years show a significantly steeper group learning curve when compared to middle aged dogs after controlling for individual initial performance. There are numerous studies suggesting that younger dogs show greater learning ability than aged dogs; however, to date there has been no lifespan studies of learning abilities in domestic dogs. Adolescence may reflect a sensitive period when quick and efficient learning to focus on task demands occurs in normal development in the domestic dog (Scott, 1958).

The dogs’ initial LEC and LFF across the first 20 trials were affected by dogs’ clicker training status, with dogs having previous experience being faster in gaining eye contact with the experimenter in the first three trials and also quicker in finding the food over the first 20 trials. The simplest explanation for this result is that clicker trained dogs were already familiar with this type of task, and that overall clicker training can improve human directed looking behavior in dogs. Alternatively, it could indicate heightened motivation (anticipation of food reward) in clicker trained dogs, rather than an overall difference in sensorimotor capabilities. Non-clicker trained dogs could have been confused and/or distracted by the presence of the clicker, which could have resulted in longer latencies to eye contact. Since the oldest age group of geriatric dogs was also the age group with the lowest number of clicker trained dogs, it could be argued that had more of these individuals been in clicker training, the observed difference between middle aged dogs and geriatric dogs may disappear. However, adolescent dogs had a similar proportion of clicker trained dogs to middle aged dogs but a higher LEC, so clicker experience cannot explain all the variation which was present. The youngest age group had the highest proportion of clicker trained dogs (around 70%); therefore we can speculate that current clicker training for eye contact in this age group could also have contributed to faster latencies to eye contact with the experimenter, and younger clicker naïve dogs may show a reduced performance in the alternating attention task.

Dogs’ reproductive status influenced their performance in LFF. Neutered dogs were faster to find dropped food over 20 trials than intact dogs. Neutering increases food motivation and decreases metabolic rate, which can lead to lower energy levels and increased risk of obesity (Duffy and Serpell, 2006; German, 2006). Therefore, it is possible that neutered dogs had a greater motivation to obtain the food than intact dogs. However, the reproductive status of the dog had no effect on selective attention, which suggests that for general measures of attention and trainability there are no differences between hormonally intact and neutered dogs.

## GENERAL DISCUSSION

We investigated the lifespan development of attentiveness of pet dogs in naturalistic situations, by developing several short simple tasks designed specifically to examine possible age effects and by measuring specific components of attention. We examined the normal rate of attention development and decline in a cross-sectional sample of pet dogs from 6 months to old age, and finally, we compared the cross-sectional developmental trajectories of the different attentional components found in dogs to the existing literature in humans. The results from experiment 1 when compared to the human literature, show a similar lack of age effects on attentional capture abilities in humans and dogs, but also reveal differences in task relevance in sustained attentional performance. In experiment 2 we found that selective attention performance in adolescent and geriatric dogs was weaker than in middle aged dogs. We suggest that a U shaped developmental pathway of selective attention may be present, if younger age groups were also examined, and based on our initial results before outliers were removed. Younger and older dogs’ performance can be explained by greater levels of distractibility, which has been attributed to weakened inhibitory control (Duchek et al., 1998).

In order to help draw comparisons across the lifespan of humans and dogs, it is necessary to establish the relationship between chronological and physiological age in both species. Patronek et al. (1997) developed a method to standardize the chronological age of dogs in terms of physiological time using human year equivalents. The relationship between human age and dog age and development cannot be described accurately with a simple linear relationship, as development is not constant over a dog’s life span. A polynomial relationship allows for the human year equivalents for dogs’ ages to be larger during growth and smaller during maturity. Using Patronek’s method, the human equivalent age ranges of the dogs in this study was 10–83 years. Thus, regarding simpler forms of attention, a rather crucial developmental stage may have been missed by only testing dogs from 6 months onward. For example, in humans, attentional capture abilities reach adult levels by age 5–7 years. In order to test this in the dogs we would have needed to test them before 3 months of age. The quadratic relationship between selective attention and age (found before outliers were removed) may have been strengthened, had we tested younger dogs. Dogs’ selective attentional and sensorimotor abilities peaked at the human equivalent of roughly 28–38 years old, which is around the same time as in human studies (20–30 years old). However, from 15 to 39 years, performance in humans was similar, with few if any differences between these age groups (Clark et al., 2006). A quadratic effect of age in dogs’ attentional control could reflect improving capabilities over the years of development followed by decline during old age. Given that the development of attentional control may be similar across humans and dogs, we can speculate that the same mechanism regulates control in both species. Indeed, recent behavioral and physiological research on impulsivity in dogs indicates this might well be the case (Miller et al., 2010; Wright et al., 2012). However, longitudinal studies are needed to validate these suggestions.

The fact that dogs of all age groups were able to improve their selective attention performance in the alternating attention task is of particular importance. Even dogs which had been previously trained to gain eye contact with their owner benefited from the training with the experimenter. This improvement may be explained by the fact that dogs do not automatically transfer training exercises/cues/commands to new trainers (strangers) and to new contexts, unless they have been specifically trained to do so (Hilliard, 2003). There are two possible explanations for the dogs performance in the selective attention measure: (1) dogs were able to improve their level of attentional control over the 20 trials through an increased ability to inhibit prepotent responses, and (2) simple conditioning led to an increased relevance of the stimulus (the experimenters face) over the repeated trials. Most likely both explanations contributed to the dogs’ performance. Instrumental conditioning can explain why previous training allowed clicker trained dogs to outperform non-clicker trained dogs in the initial three trials.

Dogs with the equivalent human age of 16–23 years (1–2 in chronological years) benefited more from eye contact training with the experimenter than middle aged dogs. Human research also points to the teenage and adolescent years as a highly important transitional phase marked by significant physical, social, cognitive, and emotional changes (Crone, 2009). Just as in humans, dogs of all ages, including dogs which were clicker trained were able to benefit from a practice period. However, it remains to be seen whether training in just one area can lead to improvements across multiple domains (emotional, intellectual and physical) as has been observed in humans (Oaten and Cheng, 2006a,b), and also whether selective attention across different contexts is correlated. A recent study on dogs discovered that individual scores were not correlated between tasks of executive control (inhibition); suggesting context has a large effect on performance in these tasks (Bray et al., 2014).

Results from experiment 2 suggest that fine sensorimotor ability and attentional control may follow similar developmental pathways. Correlational evidence from cross-sectional and longitudinal studies in humans suggests a close connection between cognitive, sensory, and sensorimotor aging (Baltes and Lindenberger, 1997; Diamond, 2000; Li and Lindenberger, 2002; Li et al., 2004; Ghisletta and Lindenberger, 2005). These factors may be influenced by a common cause, an increase in resource overlap, or a combination of both (Lindenberger et al., 2000). Future research should aim to pin-point the relative importance of these possibilities using divided attentional tasks in dogs and other species.

Other important aspects to consider when studying attention and cognition in humans and animals include the influence of gender, educational level, and current training. In humans, studies have found that specific training and educational interventions targeted at influencing the development and improvement of attentional abilities has been successful at all life stages including children and older adults (Rueda et al., 2005; Oaten and Cheng, 2006a; Tang and Posner, 2009; Mozolic et al., 2011; O’Brien et al., 2013). The training score used in this study was intended as a measure of the dogs overall educational level, and the mean number of hours spent in training per week was used to reflect dogs’ current educational participation. However, the only type of training which influenced LEC and find food was clicker training experience. Two possible explanations for clicker trained dogs’ enhanced performance when compared to non-clicker trained dogs include: (1) clicker trained dogs were already familiar with the specific training method (and perhaps the task used), and therefore were better able to generalize to new contexts and trainers (Hilliard, 2003); and (2) clicker training may help to prolong and/or improve behavior such as eye contact through increased resistance to extinction (Smith and Davis, 2008). In the current study we did not find any effects of gender on any of the components of attention or sensorimotor ability.

Finally, we need to acknowledge the limitations of a cross-sectional design as a means to examine lifespan differences in attention in dogs. Schaie (2000) emphasized the potential susceptibility of cross-sectional designs to cohort differences. In the population of pet dog Border collies used for this study there were few selection pressures, and little problems with inbreeding. Most breeding dogs were chosen on either working ability (working line) sport/agility ability (sport line), or for looks or showing ability (show line). Dogs were recruited from many different breeders, pet owners, and dog schools and care was taken that individuals tested were from as diverse a sampling population as possible. However, we cannot completely rule out the possibility that the correlations and age group means we measured may not accurately reflect true developmental trajectories. Additional research using longitudinal designs would be important to confirm our findings.

## CONCLUSION

Our study provides the first cross-sectional lifespan overview of the development and aging of attention in the pet dog. Our results reveal differences in task relevance in sustained attentional performance when watching a human or a moving object, which may be explained by different life-long learning processes about such stimuli. During the attention alternation task, we found that dogs’ selective attention and sensorimotor abilities showed differences between age group means which peaked at middle age for both, indicating some association between the two processes. The differences found in selective attention in the younger adolescent and oldest age group when compared to the middle aged could be due to greater levels of distractibility, which could indicate deficiencies in attentional control abilities. When comparing sensorimotor control in previous studies in humans and the present results found in dogs, a similar quadratic effect of age was discovered. Dogs’ attentional capture and sustained attention results also paralleled those found in humans.

The importance of taking into account the dogs’ current training status in reference to examining human directed gazing behavior should be emphasized. Clicker training experience had a significant effect on dogs’ performance in the attention alternation task. Dogs of all ages significantly improved their selective attention performance over trials, with the adolescents showing a particularly enhanced learning performance in comparison to the other age groups.

Our results complement the existing research using laboratory beagles, emphasizing the importance of the domestic dog as a model species for comparative study. Finally, this study lends support to the possibility that the development of sensorimotor and attentional control and senescence may be fundamentally interrelated in dogs as proposed in humans.

## Conflict of Interest Statement

The authors declare that the research was conducted in the absence of any commercial or financial relationships that could be construed as a potential conflict of interest.
